# Cross-Cultural Adaptation and Validation of the Portuguese Version of the Multidimensional Scale of Dating Violence 2.0 in Young University Students

**DOI:** 10.3390/healthcare12070759

**Published:** 2024-03-30

**Authors:** Lorena Tarriño-Concejero, Dalila Cerejo, Socorro Arnedillo-Sánchez, Juan Manuel Praena-Fernández, María Ángeles García-Carpintero Muñoz

**Affiliations:** 1 Faculty of Nursing, Physiotherapy and Podiatry, University of Seville, 41009 Seville, Spain; ltarrino@us.es (L.T.-C.); agcarpin@us.es (M.Á.G.-C.M.); 2 Institute of Biomedicine of Seville (IBiS/CSIC), 41013 Seville, Spain; 3 Department of Sociology, Faculty of Social Sciences and Humanities (NOVA FCSH), Interdisciplinary Centre of Social Sciences (CICS.NOVA), Nova University Lisbon (NOVA), 1069-061 Lisbon, Portugal; dalilacerejo@fcsh.unl.pt; 4 Midwifery Training Unit, Department of Materno-Fetal Medicine, Genetics and Reproduction, Hospital Universitario Virgen del Rocío, 41013 Seville, Spain; 5 Faculty of Medicine-Biostatistics Unit, University of Granada, 18016 Granada, Spain

**Keywords:** dating violence, gender-based violence, health, students, validation study

## Abstract

Background: Dating violence has become a problem of social relevance with short- and long-term health consequences. Nurses are in a privileged position to detect and address this problem in health facilities and as school nurses in schools, providing health education and detecting this violence correctly. Aim: The aim of this study was to evaluate the cross-cultural validation of the Portuguese version of the Multidimensional Scale of Dating Violence-Short (MSDV 2.0). Methods: A validation investigation was carried out in two phases: (1) cross-cultural adaptation of the items and content validation of the Portuguese version of MSDV 2.0 and (2) psychometric validation. Results: Phase (1): The items of the original version include a cross-cultural translation from Spanish to Portuguese and analysed by a group of experts in gender violence and by the authors of the original scale, then a back translation was made and again reviewed by the experts. Young university students also participated for face validity, and a pilot test was carried out. Phase (2): Confirmatory factor analysis was performed using the robust maximum-likelihood estimation method, which confirmed the five-dimensional structure, obtaining good fit rates (chi-square significance (χ^2^) = 187.860 (*p* < 0.0001); root mean square error of approximation (RMSEA) = 0.049; comparative fit index (CFI) = 0.937; Tucker–Lewis index (TLI) = 0.923). Reliability analysis indicated adequate internal consistency (Cronbach’s alpha (α) = 0.88 to 0.70). Finally, scores of the Portuguese versions MSDV 2.0 were correlated, as expected, positively with the Depression, Anxiety, and Stress Scale (DASS-21) (r = 0.36 to 0.16) and negatively with the Medical Outcomes Study Questionnaire Short Form 36, Health Survey (SF-36) (r = −0.30 to −0.14). Conclusions: To date, it is the only instrument that measures dating violence in a multidimensional way validated in the Portuguese university context.

## 1. Introduction

The study and interest in dating violence (DV) began with Makepace’s study in 1981 [1]. Then, it has been conceptualised in various ways, with no current consensus definition, directly influencing the design of instruments to measure it [2]. One of the most recent definitions is developed by the Centres for Disease Control and Prevention (CDC), 2021 [3], which defines dating violence as a type of intimate partner violence that involves physical, sexual, psychological, and stalking behaviours that may occur in person or through electronic means such as repeated text messages or posting sexual photos of a partner online without consent. It occurs among young couples or adolescents who do not have a cohabiting relationship, children, or binding economic relationships [4].

Dating violence has presented high prevalence rates in recent years and is declared a public health issue [5,6]. Victims, predominantly women, are reporting greater health problems due to these behaviours. According to the World Health Organisation (WHO) in 2021, globally, the early onset of this violence is evident: By the age of 25, one in four women aged 15 to 24 who have been in any intimate relationship will have encountered violent behaviour from an intimate partner. These findings align with a recent study conducted across all European Union countries [7].

In Portugal, studies on the prevalence of gender-based violence, particularly in intimate contexts, show that it begins to manifest itself in the younger age groups, specifically in the dating phase, with prevalence rates between 10% and 15% within age groups18 and 24 years old [8,9,10].The data from the latest study carried out in Portugal in 2024 on 6152 adolescents and young people between 11 and 21 years of age indicate that of the participants who had already been in a dating relationship (*n*=3932), 63 percent (*n*=2477) reported having experienced at least one of the victimization indicators measured. Thus, the most prevalent forms of violence include 45.5% control, 39.9% psychological violence, 20.7% social media violence, 20.4% stalking, 18.5% sexual violence, and 11% physical violence. Some of the most prevalent indicators of victimisation were 31.2% insulting during an argument, 27.8% forbidding to be with or talk to a friend or colleague, 20.4% insistently searching for one’s whereabouts, 18.9% insulting via social media/internet, and 13.3% pressuring to kiss [11].

Violence within a dating couple includes a spectrum of behaviours spanning from emotional abuse to physical aggression and/or sexual violence. Regarding attitudes toward psycho-emotional abuse, there are actions characterised by denigration and devaluation aimed at inducing feelings of insecurity. These actions include threatening to terminate the relationship, implying that another individual would be a superior partner, ridiculing or insulting one’s partner in public, or asserting that they are incompetent [12]. As for physical aggression, the behaviours include a broad spectrum, ranging from threats or environmental violence to direct physical aggression [4]. The sexual violence dimension would include forced sexual relations by the partner through coercion with emotional control tactics to forced sexual activity with or without penetration [13].

In recent years, violence through information and communication technologies (ICTs) has become more relevant. These significantly influence the social relationships of young people [14] and are a new tool for exercising violence. It can manifest attitudes of control and surveillance, which involve actions such as monitoring photos and comments uploaded on social networks, tracking friendships, or insisting on deleting content or profiles. In addition, monitoring connection duration and geolocation can establish an effective control situation [15,16].

In relation to the dynamics of DV, there are studies that indicate that it often involves mutual violence [17]. However, other studies indicate that these results may be biased because most of these instruments have a “gender blindness”. In other words, “research does not take into account the gender dimension as a significant category for the approach and interpretation of research problems, either due to lack of training, because it considers that gender is not related to this problem or for other types of reasons (including possible resistance to assuming this point of view)”, where women are the victims of this violence [18].

In general, studies indicate that young people dating abuse, both victimisation and perpetration, is associated with a range of negative short- and long-term health outcomes [19]. Within the mental and psychological sphere, it has been found that both victims and perpetrators show symptoms of depression and increased suicidal ideation and attempts across time, as well as post-traumatic stress disorder [20,21]. Mental disorders play a dual role, as both risk factors for and outcomes of DV [22]. DV has also been associated with feelings of loneliness/isolation, impaired cognitive clarity [23], self-esteem [24], increased distress, sleep disturbances [25], and a sense of identity [26].

Regarding physical health outcomes, in general, DV has strong negative implications in terms of self-rated health and physical complaints [23,25,27]. They also show a range of negative weight control behaviours [25] that affect males and females in different ways. While women present an increased body mass index [28] and risk of unhealthy weight control behaviours [29], men have an increased risk of presenting binge eating [28]. Research has also associated DV with an increase in sexual risk behaviours, like inconsistent condom use, multiple sex partners, sexting, risk of teen pregnancy, and sexually transmitted infections with greater affectation in women [21,30].

On the other hand, findings indicate that undergoing dating abuse during adolescence contributes to subsequent experiences of being victimised or perpetrating abuse by a romantic partner later [20,21,24,27]. This underscores the importance of identifying and addressing these violent behaviours from their earliest stages. In this regard, nursing plays a pivotal role, as school and community nurses can engage in educational initiatives to promote healthy egalitarian relationships within the educational setting. They can also conduct screenings to detect DV and intervene before it detrimentally impacts health [31].

Given the significant prevalence and incidence of domestic violence (DV), it is imperative to have instruments that can detect and measure these behaviours. Consequently, over the past decade, various instruments have been developed and published. These instruments encompass several dimensions, including the Dating Violence Questionnaire (DVQ) [32], Violence in Adolescents’ Dating Relationships Inventory (VADRI) [33], Measure of Adolescent Relationship Harassment and Abuse (MARSHA) [34], Teen Dating Violence: Victimisation and Perpetration Scale (TDV-VP Scale) [35], Dating Violence Questionnaire-R (DVQ-R) [36], Conflict in Adolescent Dating Relationships Inventory Short Form (CADRI-S) [37], the Conflict Tactics Scales (CTS) in its different versions, modified (CTS-M) [38] or revised (CTS-2) [39], Multidimensional Dating Violence Scale (MSDV) [12], and Multidimensional Dating Violence Scale 2.0 (MSDV 2.0) [40].

Of all these instruments, the MSDV 2.0 has proven to be a short and simple instrument that measures DV in a multidimensional way, by analysing its five dimensions: cyberbullying, control and surveillance, psycho-emotional, physical, and sexual.

### The Present Study

This study aimed to cross-culturally adapt and validate the Multidimensional Dating Violence Scale (MSDV 2.0) in Portuguese university students. The Spanish version of MSDV 2.0 (victimisation) contains 18 items that measure five dimensions of violence (cyberbullying, control and surveillance, psycho-emotional, physical, and sexual). It has shown good internal consistency (Cronbach’s alpha: 0.703 to 0.828), convergent validity, and adequate adjustment indices in confirmatory factor analysis [40]. The detection of dating violence should be a priority issue in health services, since violence negatively affects physical, mental, and sexual health, where women have higher incidence rates [41]. The WHO indicates that violence against women can be prevented. The health sector has an important role to play in providing comprehensive care. There is a need for comprehensive sex education in the curricula of young people to create healthy and safe contexts, in which young people develop, and to have sensitive, valid, and reliable measurement instruments [41].

Adhering to WHO guidelines, our study was conducted within the context of Portuguese universities—a setting where young individuals socialise, interact, and dedicate a significant portion of their time. Universities are crucial institutions for promoting health, fostering egalitarian relationships, and enhancing the well-being of both students and staff, as well as society at large. They play an active role in leading and supporting processes of social change [42,43]. Portugal is affiliated with the Ibero-American Network of Health Promoting Universities (RIUPS), which prioritises promoting health and fostering relationships based on equality and mutual respect. This commitment entails integrating health into policies in a comprehensive manner and developing healthy university plans [44].

Second, an assessment instrument has been designed to measure dating violence in a multidimensional, brief, and simple way, filling a gap in knowledge in the validation of dating violence instruments; until now, there has not been a validated instrument in Portugal with these characteristics.

Therefore, our first hypothesis postulated that the factorial structure of the MSDV 2.0 among young Portuguese university students would align with the five dimensions proposed by the original scale. Secondly, we hypothesised that higher scores on the MSDV 2.0 would correlate with poorer health status.

## 2. Materials and Methods

### 2.1. Research Design

**Figure 1:**
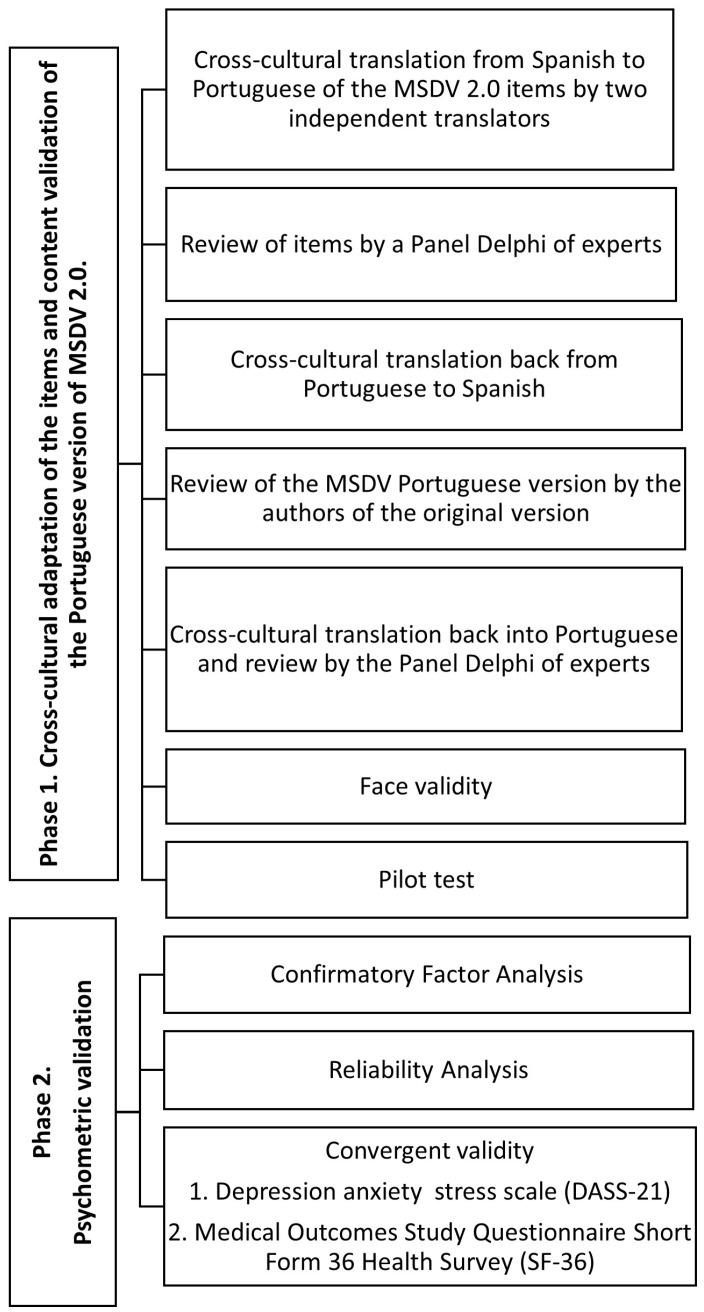
Phases of the research.

The Portuguese version of MSDV 2.0 underwent cross-cultural adaptation and validation in the Portuguese university population. The research was conducted in two phases. In the first phase, the cross-cultural adaptation of the items and content validation were carried out, followed by apparent validity and piloting. In the second phase, the psychometric properties (confirmatory factor analysis, convergent validity, and reliability analysis) were examined, testing the scale in a sample of university students. They followed the guidelines of the Consensus-based Standards for the selection of health Measurement Instruments (COSMIN) checklist [45] for this study (Figure 1). The study was conducted from July to February 2022 at the University of Social and Human Sciences of the Nova University of Lisbon (Lisbon, Portugal).

#### 2.1.1. Phase 1. Cross-Cultural Adaptation of the Items and Content Validation of the Portuguese Version of MSDV 2.0

A back translation was carried out with a critical analysis of the content for cultural adaptation. MSDV 2.0 was translated from Spanish to Portuguese by two independent translators. One of them native of Portugal and another native of Spain with a C1 level of Portuguese language, both with training in gender-based violence. The two translations were presented to a committee of experts on gender violence, made up of three researchers and professors from the National Observatory of Gender Violence of Portugal. All of them have numerous publications about DV, belong to the Council of Europe against Gender Violence, and have participated in considerable meetings to design public policies aimed at eradicating gender-based violence. 

The expert committee reviewed the dimensions and items of the MSDV 2.0 individually. After the analysis, semantic, conceptual, content, and criterion equivalence were found with the original instrument. Also, the scale reflected the most prevalent behaviours that are estimated in Portugal. After that, a single version of the scale was agreed upon, with changes in the items to adapt to the definitory elements of DV and culture in the Portuguese context, and not only a mere translation of the items. This version was translated into Spanish, by a different translator, and was reviewed again by the authors of the original version who confirmed that it preserved the semantic, conceptual, content, and criterion equivalence. It was then translated again into Portuguese and revised once again by the committee of experts. The same liker score was maintained for the Portuguese version with five response options (1: never; 2: sometimes (1 or 2 times); 3: occasionally (3–4 times); 4: repeatedly (5–10 times); 5: habitually (more than 10 times)).

Face validity was carried out to analyse the clarity, accuracy, and comprehension of the items agreed upon in the previous phase [46]. The sample included 25 university students, 15 women and 9 men with an average age of 21.8 years (SD = 1.62) participated. No errors were identified; all items were understood, clear, and accurate with mean scores of between 3.08 and 3.68, on a scale of 1 to 4, where items with means ≥3 are accepted according to the criterion proposed by Abad et al. (2011) [47]. Table 1 presents the items from the Portuguese version of MSDV 2.0.

#### 2.1.2. Phase 2. Analysis of Psychometric Properties

Psychometric properties were checked to prove that the instrument was valid and reliable. In terms of structural validity, a confirmatory factor analysis was performed. Its internal consistency and convergent validity were examined. For convergent validity purposes, we used two measures that we anticipate would correlate with the Portuguese MSDV 2.0 version: the Depression, Anxiety, and Stress Scale (DASS-21) and the Medical Outcomes Study Questionnaire Short Form 36 Health Survey (SF-36). Sociodemographic variables were also considered.

All these measures were incorporated into the final questionnaire, and a pilot test was conducted prior to proceeding with the phases of analysing the psychometric properties. The objective was to mitigate potential biases and errors in obtaining subsequent data [48]. 

### 2.2. Measures

#### 2.2.1. Demographic Variables

Sociodemographic variables studied included sex, age, residence, social/financial support (scholarships, housing), employment status and hours dedicated to work, the average duration of dating relationships, current relationship status, and cohabitation status with a partner.

#### 2.2.2. Depression, Anxiety, and Stress

This variable was evaluated using the Depression, Anxiety, and Stress Scale (DASS-21), which has been validated in Portugal [49]. The DASS-21 is a self-administered instrument comprising 21 items. This scale consists of 3 subscales that assess various areas of emotional state: depression, anxiety, and stress in a non-clinical young population. Its 21 items are evaluated according to a Likert scale, with 4 response options, from 0 to 3, where higher scores indicate poorer levels in these dimensions. The scale demonstrates good psychometric properties, with an internal consistency of 0.9. The starting hypothesis was that the higher the levels of dating violence perpetrated and suffered, the worse the levels of depression.

#### 2.2.3. Health-Related Quality of Life

This variable was assessed using the Medical Outcomes Study Questionnaire Short Form 36 Health Survey (SF-36). This scale consists of 36 items that measure 8 dimensions of health: physical function, physical role, body pain, general health, vitality, social function, emotional role, and mental health. Each dimension obtains scores between 0 and 100, where 0 is the worst state of health and 100 is the best state of health related to each of the dimensions [50]. This scale has been validated in Portugal, showing good internal consistency for its different dimensions, ranging between 0.645 and 0.875. Our hypothesis was that the higher the scores in the DV, the lower the scores in each of the dimensions of the SF-36.

### 2.3. Procedure and Participants

The data used are part of a larger dataset obtained through research on dating violence and its relationship to mental health and resilience. 

Google Form platform was used to carry out a self-administered online survey, accessible through “Inforestudante”, the university’s digital platform. The study took place from July 2021 to February 2022. 

The sample size was calculated based on the total number of students enrolled in degree programmes at the Faculty of Social and Human Sciences of Nova University of Lisbon (n = 2680) in the academic year 2021–2022. A 95% confidence level and a precision (margin of error) of 7% were used, resulting in the need to include 184 participants. Additionally, it was considered necessary to adhere to the criteria for instrument validation suggested by Mokkink et al. (2019) [45], which recommends that the sample size should comprise at least 7–10 subjects per item. Subsequently, a non-probabilistic convenience sampling method was employed to select participants from all courses, ensuring adherence to inclusion criteria such as enrolment in a degree programme at the faculty, age between 18 and 24 years old, and linguistic competence in Portuguese to adequately comprehend the instrument.

### 2.4. Data Analysis

Data analysis was conducted using RStudio version 4.1.1 with the lavaan and semPlot packages.

Descriptive statistics were used in the univariate analysis. For the quantitative variables, means, standard deviation, and percentiles were calculated. For the qualitative variables, absolute and relative frequencies and confidence intervals were calculated. 

Confirmatory factor analysis (CFA) was performed to confirm the structure of the Portuguese version in the sample. We used the robust maximum-likelihood estimation method to mitigate the possible biases that could occur in the estimates due to the observed floor effects [51], which are frequent in psychological scales that measure violence. The fit indices included were chi-square significance (χ^2^): (0≤ χ^2^ ≤df) [52]; root mean square error of approximation (RMSEA): (0 ≤ RMSEA < 0.05); comparative fit index (CFI): (0.97 ≤ CFI ≤ 1.00) [53]; and Tucker–Lewis index (TLI) (0.95 ≤ TLI ≤ 1.00) [54].

The convergent validity of the victimisation MSDV 2.0 was tested using the DASS-21 and SF-36. The starting hypothesis was that present a strong and positive relationship with DASS-21 and a strong and negative relationship with SF-36. Bivariate correlations were performed with Spearman’s rho coefficient. Previously, normality was calculated with the Kolmogorov–Smirnov test, which turned out not to follow normality. The following correlation ranges were considered: from 0.91 to 1.00 perfect; 0.76 to 0.90 very strong; 0.51 to 0.75 considerable; 0.11 to 0.50 mean; 0.01 to 0.10 weak; and 0.00 without correlation [55].

Internal consistency was determined by Cronbach’s alpha test, with an acceptable value of greater than 0.7 [56]. Ordinal coefficient alphas and McDonald’s omegas were calculated based on the polychoric correlation matrix, using the formula provided by Dominguez-Lara [57], the McDonald’s Omega and Greatest Lower Bound with psych and psychTools packages of R. Total explained variance of the common factor was obtained.

### 2.5. Ethical Considerations

This research was approved by the research support division of the Nova University of Lisbon (Lisbon, Portugal), with study code 1/CE_NOVAFCSH/2021. The entire study complied with the Declaration of Helsinki on the ethical protection and regulation of research among human beings. Participants received verbal and written information about the research (objectives, methodology, and purpose of the results). Their participation was voluntary and without financial consideration. All participants signed an informed consent form. The data were processed with strict anonymity and confidentiality. For the data analysis, only two researchers accessed the data for added security. The data were securely stored in the Department of Nursing at the University of Seville once downloaded from the Google Forms platform.

## 3. Results

### 3.1. Demographic Characteristics

The sample consisted of 206 participants, 73.31% women and 26.69% men, with a mean age of 21.10 (SD = 1.84). In addition, 92.2% lived in urban areas, 7.8% in semi-urban areas, and 22.8% worked an average of 6.25 h (SD = 12.43). All participants had been in a relationship with a mean time of 19.40 months (SD = 17.12). At the time of the study, 37.38% were in a relationship and 7.8% were cohabiting together (Table 2).

### 3.2. Confirmatory Factor Analysis

**Figure 2:**
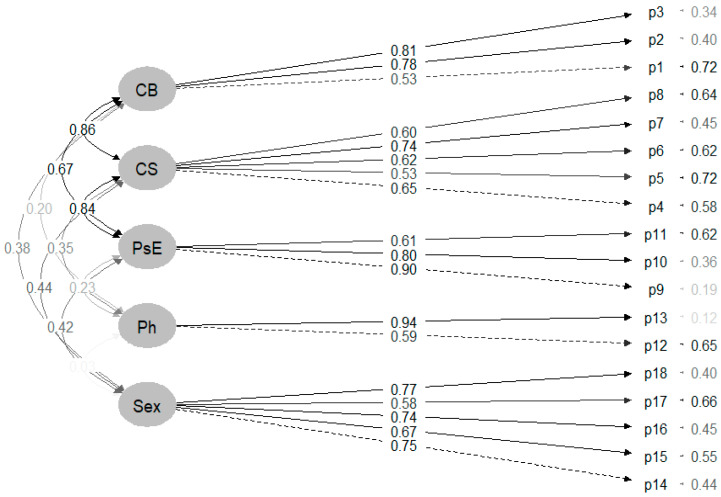
CFA diagram victimisation MSDV 2.0 Portuguese version. Note: CB: cyberbullying; CS: control and surveillance; PsE: psycho-emotional; Ph: physical; Sex: sexual.

The robust fit indices were excellent for both the victimisation subscale (χ^2^ = 187.860 (*p* < 0.0001); CFI = 0.937; TLI = 0.923; and RMSEA = 0.049 (90% CI: 0.039–0.073)). Figure 2 shows the final model selected with their factorial load. As can be seen, in the victimisation MSDV 2.0, all items have loads greater than 0.53.

### 3.3. Convergent Validity

The existence of statistically significant correlations (*p* < 0.01) was confirmed for all dimensions of the MSDV 2.0 with all the proposed dimensions of the DASS-21 scale. Positive correlations were obtained in medium degree for the dimensions of cyberbullying, control and surveillance, psycho-emotional, and sexual with the dimensions of depression, anxiety, and stress. The only dimension that showed a lower correlation was physical violence (r = 0.159–0.188, *p* < 0.01). These results confirmed the previously stated hypothesis that higher dating violence scores would correlate with higher stress, depression, and anxiety scores.

On the other hand, related to the dimensions of the SF-36, not all its dimensions were related to the different dimensions of the MSDV 2.0. 

The only dimension of the MSDV 2.0 that showed significant negative correlations to a medium degree with all the dimensions of the SF-36 was the sexual dimension (r = −0.288, *p* < 0.01; r = −0.142, *p* < 0.05).

The vitality, social function, emotional role, and mental health dimensions of the SF-36 showed significant negative correlations with all dimensions of the MSDV 2.0, except for physical violence. This last dimension showed no correlations for any of the SF-36 dimensions. Thus, confirming another of our hypotheses, in part, where higher MSDV 2.0 scores would correlate with worse health-related states measured by SF-36. The results of these correlations are given in Table 3.

### 3.4. Reliability—Internal Consistency

Considering that the confirmatory factor analysis (CFA) revealed that the MSDV 2.0 is a multidimensional scale, Cronbach’s alpha was assessed for each dimension. The reliability coefficients (Cronbach’s alpha) ranged from 0.66 to 0.81, with the lowest values observed in the physical dimension. Overall, the MSDV 2.0 demonstrated good internal consistency (Cronbach’s alpha = 0.88) (see Table 4).

### 3.5. Descriptive of Dating Violence in Young Portuguese University Students

The most prevalent dating violence (at least experienced 1–2 times) by young people was cyberbullying (63.93%), followed by psycho-emotional violence (57.46%), control and surveillance (46.02%), sexual violence (20.28%), and finally physical violence (5.55%). All behaviours measured with MSDV 2.0 were found to be more frequent in women than in men. In the analysis of differences according to gender, statistically significant variances were observed in the dimensions of cyberbullying, control, surveillance, psycho-emotional, and sexual aspects, with women being the primary targets of these violent behaviours. The only dimension that did not exhibit statistically significant differences was the physical dimension (Table 5).

## 4. Discussion

Our results suggest that the Portuguese version of the MSDV 2.0 shows favourable psychometric properties, displaying high levels of reliability and validity among young university students. The study confirmed the presence of five underlying factors within the 18 items of the scale, consistent with the version validated in the Spanish university context. 

In reference to the factor structure, our results confirm that five factors underlie dating violence, as in the original version [40]. This suggests that the Portuguese version of MSDV 2.0 is a five-dimensional measure of dating violence, reiterating the intercultural applications of the instrument. The CFA exposed through their adjustment indices (X2, CFI, TLI, and RMSEA) showed values very similar to those of the original scale, with some loads having higher values. Not only the structure of the five dimensions was confirmed, but the same items were maintained in each dimension.

In relation to convergent validity, our results coincide with previous research and the research of the original scale, in which DV was positively correlated with constructs such as depression, anxiety, and stress [50], where participants showed a higher mean in violence victimisation correlated with higher mean scores in states of anxiety, depression, and stress. In relation to the health-related quality of life, we hypothesised following the published guidelines of the WHO that higher rates of violence would correlate with worse physical, social, and emotional health states, as well as the quality of life [41]. In our study, not all dimensions of the MSDV 2.0 correlated with all dimensions of the SF-36. Thus, our results showed negative and significant correlations in the cyberbullying, control and surveillance, psycho-emotional, and sexual dimensions of the MSDV 2.0 with the dimensions that were more related to the psychological sphere of the health of the SF-36 (vitality, social function, emotional role, and mental health). The physical dimension of the MSDV 2.0 did not correlate with any of the dimensions measured by the SF-36. This could be because, as has been shown, psychological violence (cyberbullying, control and surveillance, and psycho-emotional) quadruples in prevalence rates to physical and sexual violence, and high levels of physical and sexual violence have not yet been shown to be relevant or at least the participants have not identified them as such.

As for the cultural interferences of dating violence analysed, the cross-cultural adaptation process, including back translation and critical evaluation by experts and the original scale authors, allowed for the maintenance of semantic coherence. This enables the comparison of mean scores and facilitates the drawing of certain inferences. The average item scores of the Portuguese version of the MSDV 2.0 were higher compared to the original version; minus three items showed lower means (4. Giving gifts or performing unsolicited favours/tasks, 5. Purposely passing by places where the other person is usually (home, work, bars, parties, etc., and 12. Physically assaulting someone you know) [40]. The dimensions that showed the highest prevalence in our study were cyberbullying (63.93%), followed by psycho-emotional violence (57.46%), control and surveillance (46.02%), sexual violence (20.28%), and finally physical violence (5.55%). These results are in line with the latest study conducted in 2024 on DV in Portugal [11], research conducted using the Spanish version of the MSDV 2.0 [58], and the latest nationwide survey conducted in Spain on violence against women. In relation to differences based on gender, women scored significantly higher in being victims of cyberbullying, control and surveillance, psycho-emotional, and sexual aspects. However, in the study of the Spanish version of the MSDV 2.0, the sexual dimension did not yield significant differences. Overall, these data highlight that Portuguese and Spanish cultures share many similarities in terms of dating violence, suggesting a unique opportunity for both countries to design intervention protocols collaboratively. 

In addition, it is important to point out that the scores obtained on the scale were low, which coincides with the original version and with other validation studies of instruments that measure intimate partner violence [32,34,35,36,38,59]. These low scores could potentially be attributed to the study being conducted within a university setting that prioritises the advancement of egalitarian relationships, thereby potentially influencing participants’ responses [60]. It is also possible that the young people in our study do not identify certain behaviours as violent, because they are justified under the myths of romantic love [53].

Finally, following the guidelines of the COSMIN Risk of Bias checklist [36], the quality of the instrument was independently assessed by three experts (two experts in psychometrics and one in dating violence) who had not participated in the content validation phase. Good to adequate in the properties that were developed: PROM development; content validity; structural validity; internal consistency; and hypotheses testing for construct validity (Appendix A).

### 4.1. Strengths and Limitations

This study shows the validity and reliability of a short scale that measures dating violence in a multidimensional way, the MSDV 2.0 in the Portuguese university context. To date, it is the only valid and reliable instrument that exists in this context and can be used as a screening of DV in the community context. The university environment is a cornerstone of egalitarian relationships, serving as a catalyst for social change. To achieve this, it is essential for it to be a safe space free from DV, and the instrument designed can help identify cases of DV and address them. Furthermore, our research fulfills one of the commitments of the WHO and the sustainable development goals, specifically Goal 5 (gender equality) and Goal 4 (quality education), emphasising the need for valid and reliable measurement instruments. 

The results should be interpreted with caution given the limitations of this study. The first is that the sample was for convenience, not representative of the complete Portuguese university context. Second, the scores reported by the participants were low, being able to reach floor effect in some items. But this difficulty has been evidenced in a large percentage of research in this field, as we have pointed out above [32,34,35,36,38,59]. To solve this, the CFA performed robust maximum likelihood estimation to mitigate possible biases that could occur in the estimates due to the observed floor effect, and in the internal consistency, other statistical values such as ordinal coefficient alphas and McDonald’s omegas were provided to demonstrate that the items consistently follow the same construct. Finally, it should be noted that the size and characteristics of the sample make it difficult to carry out measurement invariance analysis between men and women.

### 4.2. Involvement in Policy, Practice, and Future Research

The results of this study may be used as a first approach to developing health policies and health promotion plans in the university context, in which the research was developed. Since the percentages of DV shown are worrying, attention should be placed on the violence that is exercised through the different social networks. These data can be used to design or modify protocols against DV and to elaborate guidelines for healthy attitudes in relationships.

In practice, the Portuguese version of MSDV 2.0 is a short and easy-to-administer instrument that can be used as a screening in the university environment along with other health-related instruments. It could also prove its validity in the health field and be an instrument present in our women’s health care consultations. 

Finally, further research in the field of dating violence and health is encouraged. Since the objective of this study was the cross-cultural adaptation and validation of the Portuguese version of the MSDV 2.0 in young university students, but in its convergent validity, it has been shown that young people who present DV suffer health affectations, with a greater impact on mental health, and in a greater percentage those affected are women. A longitudinal study could be carried out with a larger and more representative sample to analyse what elements influence DV and design care strategies to help young university students have healthy relationships and therefore enjoy better physical, mental, and social health.

## 5. Conclusions

It has been shown that being a victim of DV can be a precursor to violent relationships in later stages. In addition, DV is a major public health concern, with girls being most affected by associated health issues. There is a need to develop valid and reliable instruments that detect and measure DV in a multidimensional way. Our findings suggest that the Portuguese adaptation of the MSDV 2.0 demonstrates favourable psychometric properties, showcasing high levels of reliability and validity among young university students.

The development of instruments that detect and measure DV is of crucial social significance within the sphere of healthcare sciences. These instruments provide a structured and scientific approach to assessing the prevalence and severity of DV, thereby providing enhanced comprehension of this phenomenon. These precise and reliable data concerning DV make it feasible to design and implement effective prevention and intervention strategies. Moreover, such instruments aid in identifying risk factors and protective elements associated with DV, facilitating the implementation of tailored interventions. Ultimately, they serve to raise awareness of the issue and catalyse social shifts toward healthier, violence-free relationships. 

Primary health care and school nurses have a challenge in detecting and addressing DV, since, as indicated by the WHO, nurses are in a privileged position in women’s health care. In addition to detection, nurses play a fundamental role in addressing health issues associated with DV. This involves providing emotional support, education on healthy relationships, and intervention options. Nurses are trained to deliver comprehensive care to victims of gender-based violence, addressing both the physical and psychological needs that may arise. Regarding prevention, school nurses hold a pivotal role in designing health promotion initiatives centred around fostering egalitarian relationships free of violence. Moreover, nurses play a crucial role in interdisciplinary collaboration, working closely with other healthcare professionals, social workers, and community organisations to ensure a comprehensive response to DV.

It is necessary to continue research on DV to identify additional variables related to DV and its implications on health, as well as conduct longitudinal studies to continue exploring deeper into the results obtained. 

## Figures and Tables

**Table 1:** Portuguese version of MSDV 2.0.

Portuguese Version MSDV 2.0	Spanish Version MSDV 2.0	D
Enviar insistentemente mensagens através de redes sociais (Facebook, Whatsapps, Twitter, TikTok, Snapchat, Tinder, Instagram, ou outros)	Enviar con insistencia Whatsapps, u otro tipo de mensajes por redes sociales	CB
2. Espiar a atividade nas redes socias (por exemplo ver os comentários em fotos enviadas por amigos/as para saber o que é dito o que se faz e com quem)	2. Espiar la actividad del otr@ en las redes: comentarios a fotos subidas por amig@ con el fin de saber qué dice, qué hace y con quien	CB
3. Controlar a hora em que esteve, pela última vez, ligado/a no whatsapps e/ou noutras redes sociais	3. Controlar la hora de la última conexión en whatsapps y/o redes sociales del otr@	CB
4. Dar presentes ou realizar favores/tarefas não solicitados	4. Hacer regalos o favores no solicitados	CS
5. Passar de propósito por lugares onde a outra pessoa costuma estar (casa, trabalho, bares, festas…)	5. Pasar a propósito por los lugares el/la otr@ suele estar (casa, trabajo, bares, fiesta…)	CS
6. Perguntar constantemente onde está ou o que está a fazer	6. Preguntar dónde está “cada minuto del día” y/o que está haciendo el/la otr@	CS
7. Tentar fazer com que a outra pessoa se sinta culpada por não passarem suficiente tempo juntos	7. Tratar de hacer sentir culpable el/la otr@ por no pasar suficiente tiempo juntos	PsE
8. Confirmar junto de amigos, familiares ou outros, se é verdade que a outra pessoa se encontrava onde disse ter estado ou estar	8. Comprobar por amistades, familiares u otra vía, si es cierto que el/la otro/a estaba donde decía estar	PsE
9. Relembrar algo negativo do pasado para causar dano emocional	9. Sacar a relucir algo del pasado para hacer daño	PsE
10. Culpar a outra pessoa de situações ou eventos que correram mal	10. Culparl@ de las cosas que no salen bien	PsE
11. Evitar ou negar-se a falar com a outra pessoa (por muito tempo) quando se está zangado/a	11. Evitar o negarse a hablar con la otra persona (durante mucho tiempo) cuando se está enfadado/a	PsE
12. Agredir fisicamente a alguém conhecido	12. Dañar físicamente a alguien conocido	Ph
13. Agredir fisicamente através de socos, pontapés ou estalos	13. Agredir físicamente a la otra persona de forma grave (bofetada, puñetazo)	Ph
14. Ter sexo sem consentimento explicito, ou quando a pessoa se encontrava incapaz de dar o seu consentimento	14. No solicitar el consentimiento para mantener relaciones sexuales	Sex
15. Aproveitar que a outra pessoa está sob o efeito do álcool ou outras drogas para ter práticas sexuais	15. Aprovechar que el/la otr@ está borrach@ o drogado@ para tener relaciones sexuales	Sex
16. Pedir alguma prática sexual que o outro não quisesse fazer, como a penetração, usar de objetos perigosos, ou ter práticas sexuais indesejadas com outras pessoas	16. Pedir alguna práctica sexual que el/la otr@ no deseaba hacer, como penetrar o usar objetos peligrosos, o tener relaciones no deseadas con otras personas	Sex
17. Presionar a realizar práticas sexuais sem preservativo	17 Presionar para tener prácticas sexuales sin preservativo	Sex
18. Tocar de modo explícitamente sexual sem consentimento	18. Hacer tocamientos sexuales sin que la otra persona quiera	Sex

Note: D: dimension; CB: cyberbullying; CS: control and surveillance; PsE: psycho-emotional; Ph: physical; Sex: sexual. Portuguese version of MSDV 2.0: A version adapted to the culture and defining elements of dating violence in Portugal in young people (not only the simple translation of the items from the original version, Spanish version of MSDV 2.0).

**Table 2:** Sociodemographic characteristics of participants.

Variables	Women (n = 151)	Men (n = 55)	Total (n = 206)
Age (M; SD)	20.01; SD = 1.74	20.35; SD = 2.11	20.10; SD = 1.84
Residence			
Urban (n; %)	141; 93.4%	49; 89%	190; 92.2%
Rural (n; %)	10; 6.6%	6; 11%	16; 7.8%
Social/Financial support from the university (scholarship, housing)	42; 27.8%	9; 16.4%	51; 24.8%
Work (active) (M; SD)	34 (22.5%)	13 (23.6%)	47 (22.8%)
Weekly hours (M; SD)	6.2; SD = 13.2	6.39; SD = 12.76	6.25; SD = 12.43
Average time (months) in a dating relationship (M; SD)	20.64; SD= 17.59	15.96; SD = 15.40	19.40; SD = 17.12
Dating relationships in the last year (M; SD)	1.07; SD = 0.28	1.05; SD = 0.23	1.06; SD = 0.26
Currently in a romantic relationship (Mean, SD)	50 (33.11%)	27 (49.1%)	77 (37.38%)
Living with a partner (Mean, SD)	11 (7.28%)	5 (9.1%)	16 (7.8%)

Note: n: number of participants; %: percentage; M: media; SD: standard deviation.

**Table 3:** Spearman correlation coefficients between MSDV 2.0 and DASS 21 and SF-36.

	MSDV 2.0 Cyberbullying	MSDV 2.0 Control and Surveillance	MSDV 2.0 Psycho-Emotional	MSDV 2.0 Physical	MSDV 2.0 Sexual
DASS-21 (Depression)	0.25 **	0.34 **	0.30 **	0.16 *	0.30 **
DASS-21 (Anxiety)	0.33 **	0.35 **	0.36 **	0.19 **	0.31 **
DASS-21 (Stress)	0.32 **	0.32 **	0.35 **	0.19 **	0.32 **
SF-36 (Physical function)	−0.06	−0.16 *	−0.14 *	−0.09	−0.018 *
SF-36 (Physical role)	−0.07	−0.13	−0.09	0.04	−0.25 **
SF-36 (Body pain)	−0.016 *	−0.10	−0.09	−0.06	−0.20 **
SF-36 (General health)	−0.07	−0.11	−0.09	−0.06	−0.14 *
SF-36 (Vitality)	−0.21 **	−0.24 **	−0.20 **	−0.03	−0.25 **
SF-36 (Social function)	−0.24 **	−0.22 **	−0.19 **	−0.05	−0.25 **
SF-36 (Emotional role)	−0.22 **	−0.27 **	−0.23 **	0.06	−0.29 **
SF-36 (Mental health)	−0.25 **	−0.30 **	−0.21 **	−0.07	−0.26 **

Note: * Correlation is significant at the 0.05 level (two-tailed); ** Correlation is significant at the 0.01 level (two-tailed).

**Table 4:** Reliability indexes for the Portuguese version of MSDV 2.0.

Dimensions MSDV 2.0	Cronbach’s Alpha	Ordinal Alpha	McDonald’s Omega	Greatest Lower Bound	Explained Variance
Cyberbullying	0.74	0.79	0.76	0.77	0.76
Control and surveillance	0.76	0.83	0.77	0.81	0.42
Psycho-emotional	0.81	0.86	0.82	0.85	0.61
Physical	0.70	-	0.82	-	0.73
Sexual	0.81	0.91	0.83	0.87	0.51
Total	0.88	0.9	0.91	0.93	-

**Table 5:** Descriptive of the items and dimensions of the Portuguese version of MSDV 2.0.

Items	Total (n = 206)	Women (n = 151)	Men (n = 55)	*p* *	Dimensions	Score Range	Total (n = 206)	Women (n = 151)	Men (n = 55)	*p* *	Percentage of Youth Who Experienced Dating Violence (At Least 1–2 Times) (%)
	Mean (SD)	Mean (SD)	Mean (SD)				Mean (SD)	Mean (SD)	Mean (SD)		
Sending messages insistently through social networks (Facebook, WhatsApp, Twitter, TikTok, Snapchat, Tinder, Instagram, or others)	2.77 (1.43)	2.85 (1.41)	2.55 (1.47)	0.154	Cyberbullying	3–15	7.47 (3.43)	7.89 (3.47)	6.31 (3.08)	0.003	
2. Spy on social media activity (e.g., view comments on photos sent by friends to find out what is said, what is done, and with whom)	2.33 (1.39)	2.51 (1.40)	1.82 (1.20)	<0.001							63.93
3. Control the time you were last connected on WhatsApp and/or other social networks	2.37 (1.43)	2.52 (1.45)	1.95 (1.28)	0.008							
4. Giving gifts or performing unsolicited favours/tasks	1.91 (1.12)	2.05 (1.19)	1.55 (0.78)	0.010	Control and surveillance	5–25	9.65 (4.25)	10.20 (4.48)	8.14 (3.08)	0.004	46.02
5. Purposely passing by places where the other person is usually (home, work, bars, parties, etc.)	1.69 (1.08)	1.79 (1.07)	1.42 (1.05)	0.003							
6. Constantly asking where you are or what you are doing	2.28 (1.35)	2.34 (1.37)	2.11 (1.30)	0.245							
7. Trying to make the other person feel guilty for not spending enough time together	2.34 (1.46)	2.50 (1.49)	1.91 (1.25)	0.011							
8. Confirm with friends, family, or others if it is true that the other person was where they said they had been or was	1.42 (0.87)	1.52 (0.95)	1.16 (0.53)	0.006							
9. Remembering something negative from the past to cause emotional damage	2.29 (1.39)	2.53 (1.44)	1.62 (0.93)	<0.001	Psycho-emotional	3–15	6.79 (3.51)	7.34 (3.63)	5.29 (2.65)	<0.001	
10. Blaming the other person for situations or events that went wrong	2.25 (1.38)	2.44 (1.43)	1.73 (1.08)	<0.001							57.46
11. Avoiding or refusing to talk to the other person (for too long) when you are angry	2.26 (1.34)	2.38 (1.38)	1.95 (1.19)	0.043							
12. Physically assaulting someone you know	1.04 (0.30)	1.06 (0.35)	1.00 (0.00)	0.173	Physical	2–10	2.17 (0.70)	2.20 (0.79)	2.10 (0.37)	0.927	5.55
13. Physically assault by punching, kicking, or popping	1.13 (0.49)	1.14 (0.53)	1.11 (0.37)	0.944							
14. Having sex without explicit consent, or when the person was unable to give their consent	1.24 (0.61)	1.32 (0.68)	1.00 (0.00)	<0.001	Sexual	5–25	6.66 (2.94)	7.26 (3.25)	5.13 (0.39)	<0.001	
15. Taking advantage of the fact that the other person is under the influence of alcohol or other drugs to have sexual practices	1.14 (0.52)	1.19 (0.59)	1.02 (0.14)	0.026							
16. Asking for some sexual practice that the other person did not want to do, such as penetration, using dangerous objects, or having unwanted sexual practices with other people	1.37 (0.84)	1.49 (0.95)	1.054 (0.19)	<0.001							20.28
17. Trying to have sex without a condom	1.41 (0.86)	1.54 (0.96)	1.05 (0.23)	<0.001							
18. Sexually explicit touching without consent	1.50 (0.98)	1.68 (1.08)	1.02 (0.14)	<0.001							

Note: * Mann–Whitney U test (*p* < 0.05).

## Data Availability

Data are contained within the article.

## Appendix A. COSMIN Risk of Bias Checklist. Evaluating the Portuguese Version of MSDV 2.0

**Psychometric Property**		**Score**		
1. PROM development		Rater 1	Rater 2	Consensus
1.a	PROM design	V	V	V
1.b	Cognitive interview study or another pilot test	A	A	A
	TOTAL Lowest score of items 1.a–1.b	A	A	A
2. Content validity		Rater 1	Rater 2	Consensus
2.a	Asking patients about relevance	V	V	V
2.b	Asking patients about comprehensiveness	V	V	V
2.c	Asking patients about comprehensibility	V	V	V
2.d	Asking professionals about relevance	V	V	V
2.e	Asking professionals about comprehensiveness	V	V	V
	TOTAL Lowest score of items 2.a–2.e	V	V	V
3. Structural validity		Rater 1	Rater 2	Consensus
3.1	For CTT: Was exploratory or confirmatory factor analysis performed?	A	V	A
3.2	For IRT/Rasch: does the chosen model fit to the research question?	V	V	V
3.3	Was the sample size included in the analysis adequate?	A	A	A
3.4	Were there any other important flaws?	A	A	A
	TOTAL Lowest score of items 1–4	A	A	A
4. Internal consistency		Rater 1	Rater 2	Consensus
4.1	Was an internal consistency statistic calculated for each unidimensional (sub)scale separately?	V	V	V
4.2	For continuous scores: Was Cronbach’s alpha or omega calculated?	V	V	V
4.3	For dichotomous scores: Was Cronbach’s alpha or KR-20 calculated?	V	V	V
4.4	For IRT-based scores: Was standard error of the theta (SE (θ)) or reliability coefficient of estimated latent trait value (index of (subject or item) separation) calculated?			
4.5	Were there any other important flaws?	V	V	V
	TOTAL Lowest score of items 1–5	V	V	V
5. Cross-cultural validity/measurement invariance		Rater 1	Rater 2	Consensus
5.1	Were the samples similar for relevant characteristics except for the group variable?	A	A	A
5.2	Was an appropriate approach used to analyse the data?	V	A	A
5.3	Was the sample size included in the analysis adequate?	A	A	A
5.4	Were there any other important flaws?	V	V	V
	TOTAL Lowest score of items 1–4	A	A	A
6. Reliability		NT		
7. Measurement error		NT		
8. Criterion validity		NT		
9. Hypotheses testing for construct validity				
9a. Comparison with other outcome measurement instruments (convergent validity)		Rater 1	Rater 2	Consensus
9.a.1	Is it clear what the comparator instrument(s) measure(s)?	V	V	V
9.a.2	Were the measurement properties of the comparator instrument(s) adequate?	A	A	A
9.a.3	Was the statistical method appropriate for the hypotheses to be tested?	V	V	V
9.a.4	Were there any other important flaws?	A	V	A
	TOTAL Lowest score of items 1–4	A	A	A
9b. Comparison between subgroups (discriminative or known-groups validity)		NT		
10. Responsiveness		NT		
Score: V: very good; A: adequate; D: doubtful; I: inadequate; N: not applicable. Not rated (NT): Only those parts of the boxes need to be completed for which its psychometric property has been realised.				
